# Visual self-assessment of dental appearance: A comparison of mirror types and digital reflection via smartphone front facing camera

**DOI:** 10.1371/journal.pone.0345596

**Published:** 2026-03-20

**Authors:** Al Imran Shahrul, Asma Ashari, Ratnah Thevi Subramanam

**Affiliations:** 1 Department of Family Oral Health, Faculty of Dentistry, Universiti Kebangsaan Malaysia, Kuala Lumpur, Malaysia; 2 Dental unit, UKM Specialist Children’s Hospital, Universiti Kebangsaan Malaysia, Kuala Lumpur, Malaysia; Universiti Sains Malaysia, MALAYSIA

## Abstract

**Introduction:**

In dental practice, patients often evaluate their own dental attractiveness using mirrors or smartphone digital reflections. This study aimed to investigate how mirror size, shape, magnification and smartphone-based digital reflection influence self-perceived dental attractiveness.

**Materials and methods:**

This was a prospective cross-sectional study. Eighty-three participants assessed their dental attractiveness using a randomised sequence of reflective devices, including mirrors of different sizes (full-length, medium, small, standard pocket), a magnified pocket mirror, a tooth-shaped mirror, and a smartphone front-facing camera. Ratings were recorded on a 10-point visual analogue scale. Data were analysed using repeated-measures ANOVA with Bonferroni-adjusted post hoc comparisons. Intra-rater reliability was evaluated in a subset of participants using the intraclass correlation coefficient (ICC).

**Results:**

Repeated-measures ANOVA indicated a significant overall effect of device type on dental attractiveness (F(4.62, 378.83) = 15.57, p < 0.001). Post hoc analyses showed no significant differences among full-length, medium, small, and standard pocket mirrors, or between these mirrors and the smartphone reflection (all p > 0.05). The magnified pocket mirror and tooth-shaped mirror were rated significantly lower than other devices (p < 0.001). Intra-rater reliability was generally poor (ICC < 0.35), with only the medium-size and tooth-shaped mirrors showing moderate agreement (ICC = 0.59 and 0.63, respectively).

**Conclusion:**

Reflective device type influences self-perceived dental attractiveness. Standard mirrors of different sizes and smartphone reflections produced similar and generally favourable ratings. In contrast, magnified and tooth-shaped mirrors yielded lower ratings. Poor intra-rater reliability suggests that self-assessment based on reflection alone is inconsistent.

## Introduction

In clinical dental practice, a patient’s perception of their dental appearance plays an important role in treatment acceptance and overall satisfaction with outcomes [1,2]. Reflective devices are routinely used by dentists to help patients visualise their teeth and smile, support explanation of aesthetic concerns, and evaluate treatment outcomes. As such, reflections function not only as tools for self-assessment but also as key aids in clinician–patient communication regarding aesthetic goals and results.

Mirrors are ubiquitous in everyday life and are commonly assumed to provide a direct and accurate representation of appearance [3]. However, mirrors differ in size, shape, and optical properties, and these characteristics can influence visual perception [4,5]. In both everyday and clinical contexts, mirrors can be categorised by size (such as full-length or wall-mounted mirrors, medium tabletop mirrors, and small hand-held or pocket mirrors), by shape (most commonly rectangular, square, or round), and by optical features, including the presence of magnification.

Despite their familiarity, research in visual perception has shown that people often reason inaccurately about mirror reflections. Bertamini et al. have identified several systematic errors in how individuals interpret reflections on planar mirrors. One commonly observed error is the belief that a reflection would become visible before reaching the near edge of a mirror when approaching it from the side, reflecting a misunderstanding of reflection geometry [6–8]. Another well-documented phenomenon is the “Venus effect”, in which observers assume that what they see in a mirror is the same as what the reflected person sees, thereby neglecting the role of viewpoint [9].

Further misconceptions relate to the perceived size of reflections. Individuals frequently overestimate the size of their reflected face, believing it to be similar in size to the actual face [8,10,11]. In addition, many people incorrectly assume that their reflection becomes smaller as they move further away from a mirror, whereas the size of the projected image on a plane mirror remains constant regardless of viewing distance [10]. Other errors include the belief that one can simultaneously see oneself reflected in multiple mirrors mounted on the same flat wall, and a tendency to misjudge the location of an object’s projection on a mirror surface by biasing it towards its virtual position [12]. Collectively, these findings indicate that people often fail to appreciate the role of the observer’s viewpoint when interpreting mirror reflections.

In addition to mirrors, smartphones are increasingly used for self-viewing [13]. The front-facing camera allows individuals to observe their appearance via a digital screen, and this form of digital reflection has become routine in daily life. Unlike mirrors, smartphone images are influenced by camera optics and digital image processing, which may alter nasal proportions [14–16], contrast, and visual detail [17,18]. However, the impact of smartphone-based digital reflections on dental self-assessment has not been well explored.

Existing literature from facial aesthetics and plastic surgery suggests that altered visual representations, such as unfamiliar image formats [19,20], can influence satisfaction with appearance even when objective outcomes are acceptable. However, these studies largely focus on facial appearance or photographic images rather than the effect of different reflective devices themselves. Within dentistry, research has primarily relied on photographs or clinician-based assessments, with limited attention to how patients perceive their own dental attractiveness when viewing different mirrors or digital reflections.

Therefore, a clear gap exists in the dental literature regarding the influence of commonly used reflective devices on self-perceived dental attractiveness. This prospective cross-sectional study aimed to evaluate how mirrors of different sizes, shapes, and magnification, as well as a smartphone front-facing camera, affect perceived dental attractiveness.

## Materials and methods

### Study design

This was a prospective cross-sectional study. Ethical approval was obtained from the institution ethical review board (Ref no: JEP-2025–207).

### Reflective devices (mirror types and digital reflections)

Six types of mirrors were utilised in this study. These included: (1) a full-length mirror (94 × 34 cm); (2) a medium-sized mirror (48 × 38 cm); (3) a small mirror (24.5 × 19.5 cm); (4) a dual-sided square pocket-size mirror (6.8 × 6.8 cm), featuring a standard reflective surface on one side and a magnified mirror on the reverse; and (5) a handheld tooth-shaped mirror, approximately 5.5 × 3.5 cm in size.

All mirrors were constructed with clear glass and enclosed in lightweight black plastic frames, except for the tooth-shaped mirror, which was made of white plastic.

For the digital reflection condition, the smartphone front-facing camera of an iPhone 12 Pro (Apple Inc., California, USA) was used. This device features a 12-megapixel TrueDepth camera with an ƒ/2.2 aperture and a wide-angle field of view equivalent to approximately 23 mm. The camera includes software-based image stabilisation. During the study, the camera was operated in selfie video mode, with no filters applied and the screen brightness standardised at 50%.

### Sample size

The sample size was calculated using G*Power version 3.1 (Heinrich Heine Universität Düsseldorf, Germany), based on an effect size of 0.15, a power of 0.95, and an alpha level of 0.05. The calculation assumed one group with seven repeated measurements. The minimum required sample size was 68 participants. To account for potential dropouts or incomplete questionnaires, the final sample size was increased to 83 participants.

### Participant recruitment

Participants were recruited from the members of the Faculty of Dentistry, Universiti Kebangsaan Malaysia. Inclusion criteria included a person aged 18 years or older. Exclusion criteria were the presence of any visual impairment that could affect visual perception or subject undergoing any active dental treatment that might influence the appearance of their teeth or alter their perception of dental aesthetics. Convenience sampling was employed, whereby all eligible students and support staff were approached, informed about the purpose of the study, and invited to participate. Written informed consent was obtained from all participants prior to their involvement.

### Randomisation

Device presentation order was determined using simple randomisation. Each mirror type and the smartphone-based digital reflection were assigned a unique numerical identifier. An online random sequence generator (Random.org; Randomness and Integrity Services Ltd., Ireland) was used to generate a unique presentation order for each participant immediately prior to data collection.

### Data collection

The assessment environment was standardised for all participants. All assessments were conducted in a clinical setting under consistent neutral white ceiling lighting, with no additional or directional light sources introduced. All mirrors were disinfected with alcohol-based wipes between participants, and any visible debris or marks were removed using a microfibre cloth prior to each assessment.

The full-length and medium-size mirrors were positioned vertically, with a standardised viewing distance of approximately 2 feet. The remaining mirrors were placed on a tabletop surface, and participants approached each device sequentially according to the randomised order assigned prior to assessment.

For the tabletop mirrors and the smartphone front-facing camera, participants were permitted to hold the device at a comfortable self-selected distance. They were allowed to observe their dental appearance for as long as they wished before proceeding to the next device. Following each viewing, participants completed a questionnaire assessing perceived dental attractiveness.

The use of a self-selected viewing distance for handheld mirrors and the smartphone front-facing camera was informed by pilot testing. When a fixed viewing distance was applied, participants reported difficulty assessing their dental appearance due to the limited field of view of smaller reflective surfaces and differences in visual acuity. Allowing a self-selected distance improved visual clarity and more closely reflected typical clinical and everyday usage. In contrast, a standardised viewing distance was maintained for the full-length and medium-size mirrors, which are commonly wall-mounted in clinical and domestic settings.

### Questionnaire

The questionnaire was adapted from the instrument developed by Yuen et al. (2023) and was administered in Malay [21]. The original questionnaire had previously undergone forward and backward translation, as well as validation in the Malay language. Prior to the present study, the adapted version was reviewed by two orthodontists to assess its clinical relevance, clarity, and suitability for the study population. Feedback was also sought from the adapted questionnaire developer, and minor refinements were made accordingly. The questionnaire was subsequently pilot-tested among five participants who were not included in the main study to evaluate clarity and comprehensibility. No further modifications were required.

Basic demographic information, including name, age, and ethnicity, was also collected. For each condition, participants rated their dental appearance using a visual analogue scale ranging from 0 (least attractive) to 10 (most attractive) [22]. Once a session with a particular mirror or the smartphone was completed, participants were not permitted to return to any previous viewing device.

### Reliability

After two weeks, 10% of the participants were asked to repeat the same assessment to evaluate intra-rater reliability.

### Statistical analysis

Data were analysed using SPSS version 26.0 (IBM Corp., Armonk, NY, USA). Descriptive statistics were calculated for each mirror type and the digital reflection. A repeated measures ANOVA was performed to assess differences in perceived dental appearance. Post hoc pairwise comparisons were conducted using the Bonferroni correction. A p-value of less than 0.05 was considered statistically significant. Intra-rater reliability was assessed using the Intraclass Correlation Coefficient (ICC) [14].

### Results

### Participant demographics

A total of 83 participants completed the study. The median age was 24.0 years (IQR = 18.0). The sample consisted of 69 females (83.1%) and 14 males (16.9%). In terms of ethnicity, 56.6% identified as Malay, 31.3% as Chinese, 9.6% as Indian, and 2.4% as other ethnic backgrounds (Table 1).

**Table 1:** Participant Demographics.

Variables	N (%)	Median (IQR)
Age		24.00 (18.00)
Gender		
Male	14 (16.9)	
Female	69 (83.1)	
Ethnicity		
Malay	47 (56.6)	
Chinese	26 (31.3)	
Indian	8 (9.6)	
Others	2 (2.4)	

### Descriptive ratings of dental attractiveness

The medium-size mirror had the highest mean attractiveness score (mean = 7.675, 95% CI: 7.327 to 8.023), followed by the full-length mirror (mean = 7.241, 95% CI: 6.781 to 7.701), and the small-size mirror (mean = 7.072, 95% CI: 6.620 to 7.524). Lower scores were observed for the pocket-size mirror with magnification (mean = 5.494, 95% CI: 4.979 to 6.009) and the handheld tooth-shaped mirror (mean = 5.747, 95% CI: 5.249 to 6.244) (Table 2).

**Table 2:** Descriptive Statistics and Repeated Measures ANOVA Results for Dental Attractiveness Scores by Mirror Type and Digital Reflection.

Mirror Types and Digital Reflections	Mean	95% CI	F (df1, df2)	p-value
Full-length Mirror	7.241	6.781, 7.701	15.570 (4.620, 378.833)	< 0.001
Medium-size Mirror	7.675	7.327, 8.023		
Small-size Mirror	7.072	6.620, 7.524		
Pocket-size Mirror (Standard)	6.843	6.451, 7.236		
Pocket-size Mirror (Magnification)	5.494	4.979, 6.009		
Handheld Tooth-shape Mirror	5.747	5.249, 6.244		
Smartphone Front facing camera	6.819	6.388, 7.251		

### Comparison of mirror types and digital reflections

The repeated measures ANOVA revealed a statistically significant difference in perceived dental attractiveness scores across the different mirror types and digital reflection methods (Table 3), F(4.620, 378.833) = 15.570, p < 0.001.

**Table 3:** Post Hoc Pairwise Comparisons of Mirror Types and Digital Reflection on Dental Attractiveness Scores (Bonferroni Adjusted).

Comparison	Mean Diff. (95% CI)	P
Full-length Mirror – Small-size Mirror	0.169 (−0.612, 0.949)	> 0.95
Full-length Mirror – Handheld Tooth-shape Mirror	1.494 (0.388, 2.600)	0.001*
Full-length Mirror – Pocket-size Mirror (Standard)	0.398 (−0.511, 1.307)	> 0.95
Full-length Mirror – Pocket-size Mirror (Magnification)	1.747 (0.634, 2.860)	<0.001*
Full-length Mirror – Medium-size Mirror	−0.434 (−1.224, 0.357)	> 0.95
Full-length Mirror – Smartphone Front facing camera	0.422 (−0.458, 1.302)	> 0.95
Small-size Mirror – Handheld Tooth-shape Mirror	1.325 (0.450, 2.200)	<0.001*
Small-size Mirror – Pocket-size Mirror (Standard)	0.229 (−0.511, 0.969)	> 0.95
Small-size Mirror – Pocket-size Mirror (Magnification)	1.578 (0.519, 2.637)	<0.001*
Small-size Mirror – Medium-size Mirror	−0.602 (−1.372, 0.167)	0.340
Small-size Mirror – Smartphone Front facing camera	0.253 (−0.714, 1.220)	> 0.95
Handheld Tooth-shape Mirror – Pocket-size Mirror (Standard)	−1.096 (−1.861, −0.331)	<0.001*
Handheld Tooth-shape Mirror – Pocket-size Mirror (Magnification)	0.253 (−0.623, 1.129)	> 0.95
Handheld Tooth-shape Mirror – Medium-size Mirror	−1.928 (−2.851, −1.005)	<0.001*
Handheld Tooth-shape Mirror – Smartphone Front facing camera	−1.072 (−2.042, −0.102)	0.018
Pocket-size Mirror (Standard)– Pocket-size Mirror (Magnification)	1.349 (0.459, 2.240)	<0.001*
Pocket-size Mirror (Standard)– Medium-size Mirror	−0.831 (−1.509, −0.154)	0.005
Pocket-size Mirror (Standard)– Smartphone Front facing camera	0.024 (−0.764, 0.812)	> 0.95
Pocket-size Mirror (Magnification)– Medium-size Mirror	−2.181 (−3.049, −1.312)	<0.001*
Pocket-size Mirror (Magnification)– Smartphone Front facing camera	−1.325 (−2.368, −0.283)	0.003*
Medium-size Mirror – Smartphone Front facing camera	0.855 (0.091, 1.620)	0.015*

* Statistically significant differences (p < 0.05).

Post hoc Bonferroni-adjusted pairwise comparisons showed that the Medium-size Mirror received significantly higher attractiveness ratings than the Pocket-size Mirror (Standard) (mean difference = 0.831, 95% CI: 0.154 to 1.509, p = 0.005). The Pocket-size Mirror (Standard) was also rated significantly higher than the Pocket-size Mirror (Magnification) (mean difference = 1.349, 95% CI: 0.459 to 2.240, p < 0.001). In addition, the Pocket-size Mirror (Magnification) and the Handheld Tooth-shaped Mirror were consistently rated significantly lower than the Full-length Mirror, Medium-size Mirror, Small-size Mirror, and Smartphone Front-facing Camera (all p < 0.05). The Smartphone Front-facing Camera received significantly higher ratings than both the Pocket-size Mirror (Magnification) (mean difference = 1.325, 95% CI: 0.283 to 2.368, p = 0.003) and the Handheld Tooth-shaped Mirror (mean difference = 1.072, 95% CI: 0.102 to 2.042, p = 0.018).

In contrast, no statistically significant differences were observed between the Full-length Mirror, Small-size Mirror, and Pocket-size Mirror (Standard) (all p > 0.95). Similarly, the Smartphone Front-facing Camera did not differ significantly from the Full-length Mirror, Medium-size Mirror, Small-size Mirror, or Pocket-size Mirror (Standard) (all p > 0.05). No significant differences were identified between the Small-size Mirror and the Medium-size Mirror (p = 0.340), or between several other mirror combinations where confidence intervals crossed zero, indicating comparable perceptions of dental attractiveness across these devices.

### Intra-rater reliability

Overall, poor intra-rater reliability was observed for most reflective devices (Table 4). However, the handheld tooth-shaped mirror and the medium-size mirror demonstrated moderate agreement, with intraclass correlation coefficients of 0.633 and 0.590, respectively.

**Table 4:** Intra-Rater Reliability of Dental Attractiveness Scores by Mirror Type and Digital Reflection.

Mirrors	ICC (95% CI)	Strength of Agreement
Full-length Mirror	0.091 (−0.214, 0.571)	Poor
Medium-size Mirror	0.590 (−0.125, 0.893)	Moderate
Small-size Mirror	0.050 (−0.674, 0.674)	Poor
Pocket-size Mirror (Standard)	−0.315 (−0.783, 0.395)	Poor
Pocket-size Mirror (Magnification)	−0.023 (−0.486, 0.570)	Poor
Handheld Tooth-shape Mirror	0.633 (0.038, 0.902)	Moderate
Smartphone Front facing camera	−0.320 (−0.962, 0.461)	Poor

## Discussion

### Summary of the results

This study demonstrated that perceived dental attractiveness varied according to the type of reflective device used, with a significant overall effect of viewing modality observed in the repeated measures ANOVA. When mirror size was considered, no significant differences were found among the Full-length, Medium-size, Small-size, and Pocket-size Mirrors (Standard), indicating that mirror size alone did not substantially influence perceived dental attractiveness. Although the Medium-size Mirror achieved the highest mean attractiveness score, this difference was not statistically significant compared with the other non-magnified mirrors.

In contrast, magnification had a clear effect on perception. The Pocket-size Mirror with magnification was consistently rated significantly lower than the Pocket-size Mirror (Standard) and all other non-magnified devices, suggesting that magnification negatively influenced perceived dental attractiveness.

Mirror shape also appeared to influence perception. The Handheld Tooth-shaped Mirror, which differs from the rectangular or square mirrors used in the other devices, received significantly lower ratings compared with most other reflective devices. This finding indicates that unconventional mirror geometry may adversely affect self-perception of dental attractiveness.

When comparing digital and non-digital reflections, the Smartphone Front-facing Camera showed no significant differences in perceived dental attractiveness compared with non-magnified physical mirrors, suggesting that digital reflection provides a broadly comparable perception of dental attractiveness.

Intra-rater reliability was generally poor across most devices, with only the Medium-size Mirror and the Handheld Tooth-shaped Mirror demonstrating moderate agreement. The remaining devices showed poor reliability, indicating substantial variability in repeated self-assessments regardless of mirror size or modality.

### Interpretation

The absence of significant differences among the full-length, medium-size, small-size, and standard pocket mirrors suggests that mirror size alone does not substantially influence perceived dental attractiveness. One possible explanation is that the visual geometry and proportions of the dentition remain consistent across different mirror sizes [3]. Although the extent of surrounding facial context varies, this does not appear to meaningfully affect dental-focused self-assessment. It is likely that participants directed their visual attention primarily towards their teeth rather than the broader facial appearance, resulting in comparable ratings across these mirror sizes.

In contrast, magnification emerged as an influential factor. Despite having a similar physical size to the standard pocket mirror, the magnified mirror consistently received lower ratings. This suggests that increased visual detail, rather than mirror size per se, may amplify perceived imperfections and negatively affect self-perception.

Mirror shape also appeared to influence perception. The handheld tooth-shaped mirror, which differs in geometry from conventional rectangular or square mirrors, was rated significantly lower than most other devices. While the precise mechanism was not measured, the unusual and asymmetric shape may provide a less intuitive visual reference, potentially interfering with proportional interpretation of the dentition. This interpretation remains hypothetical and should be explored further in future work.

Digital reflection using the smartphone front-facing camera did not differ significantly from non-magnified physical mirrors, despite differences in image processing, colour rendering, and screen-based display. This suggests that, at a group level, digital reflections may convey a perception of dental attractiveness comparable to that of conventional mirrors, even when the image is not mirror-reversed as in photographs. Software enhancement or colour tone variation alone does not appear sufficient to systematically alter self-perception in this context.

A key finding of this study was the generally poor intra-rater reliability observed across most reflective devices, including mirrors of varying sizes, magnified mirrors, and the digital reflection. This indicates that reflective-based self-assessment of dental attractiveness is inherently unstable over time, even when dental features remain unchanged. Although the Medium-size Mirror and the Handheld Tooth-shaped Mirror demonstrated moderate agreement, overall reliability remained limited. These findings suggest that reflective devices—whether optical or digital—may not be ideal tools for repeated or longitudinal assessment of dental attractiveness.

### Comparison to other studies

To date, no published studies have directly examined the effect of different mirror types or digital reflection devices on self-perceived dental attractiveness. However, relevant insights can be drawn from studies in related fields, where the influence of image familiarity and reflection format on self-evaluation has been explored.

Al-Bitar et al. (2023) [19] demonstrated that individuals tend to prefer their mirror-reversed facial images over true, non-reversed photographs, a phenomenon attributed to perceptual familiarity. In contrast, observers evaluating others tended to prefer the true image. The authors highlighted how even minor facial asymmetries may appear exaggerated when image orientation is altered, leading to increased dissatisfaction when individuals are exposed to unfamiliar representations of themselves. These findings are relevant to the present study, as certain reflective devices—such as magnified pocket mirrors or tooth-shaped mirrors—may present dental features in a manner that participants are unaccustomed to, potentially contributing to lower attractiveness ratings.

In dentistry, Babeer et al. (2021) [23] showed that small, controlled alterations in the vertical position of anterior teeth had a marked effect on perceived smile attractiveness. Their findings illustrate that subtle visual changes can significantly influence aesthetic judgements. While that study focused on anatomical modifications rather than reflective devices, it supports the broader notion that increased visibility of detail—such as that introduced by magnification—may heighten sensitivity to minor features and negatively affect aesthetic perception.

### Generalisability

The findings of this study are applicable to the specific mirror types and smartphone model evaluated. The mirrors selected were based on those most commonly used in the authors’ faculty dental clinic, and results may differ with mirrors of other sizes, shapes, or reflective properties that were not tested.

Similarly, mirror shape may affect perception. This study examined predominantly rectangular and square mirrors, along with a tooth-shaped handheld mirror. Other commonly available shapes, such as circular or oval mirrors, were not assessed and may produce different perceptual effects due to variations in visual framing and symmetry.

The generalisability of findings related to digital reflection is also limited by the smartphone model used [15]. The front-facing camera employed a wide-angle lens equivalent to 23 mm, which is known to introduce geometric distortion and may alter perceived dental proportions and facial features [16,17]. Different smartphones use lenses with varying focal lengths and optical characteristics, which may lead to different self-assessment outcomes. In addition, smartphone images undergo digital processing, including adjustments to contrast, sharpness, brightness, and, in some cases, facial enhancement. These processing algorithms vary across manufacturers and software versions.

The sampling strategy further limits generalisability. Although both students and staff were included, the majority of participants were undergraduate dental students from a dental faculty, a group likely to have heightened awareness of dental aesthetics and increased sensitivity to subtle visual differences. This professional exposure may have amplified perceptual variations between reflective devices. Moreover, the sample was predominantly female, which may have introduced additional bias, as females have been reported to be more attentive to facial and dental appearance than males [24].

### Limitations

Several methodological limitations should be considered when interpreting the findings of this study. First, a self-selected viewing distance was permitted for certain reflective devices, particularly handheld mirrors and the smartphone front-facing camera. While this approach improved visual clarity and more closely reflected real-world usage, it reduced experimental standardisation and may have introduced inter-participant variability. Differences in viewing distance and the amount of time spent assessing each device may have influenced perceived dental attractiveness.

Second, the assessment relied on a single quantitative outcome—participants’ ratings of dental attractiveness—without incorporating qualitative feedback. As a result, the study was unable to capture participants’ subjective reasons for rating differences between reflective devices, such as perceived distortion, clarity, comfort, or familiarity. Incorporating qualitative components in future research could provide deeper insight into how and why different reflective devices influence self-perception.

A further major limitation was the predominantly poor intra-rater reliability observed across most devices. Intra-rater reliability was assessed in a relatively small subsample, which may have limited the precision of the ICC estimates and reduced the ability to detect stable agreement. In addition, the use of a 10-point visual analogue scale may have contributed to variability, as such scales depend heavily on subjective judgement and may be interpreted inconsistently across repeated assessments. Fluctuations in attention, mood, or visual focus may also have influenced repeated self-ratings of dental attractiveness.

Given the observed poor reliability, the findings should be interpreted primarily in terms of relative differences between reflective devices at a group level rather than absolute numerical scores. The results highlight the need for further investigation of the reliability of reflection-based self-assessment using larger sample sizes, repeated assessments, and more anchored or categorical rating systems.

### Clinical implication

The findings highlight several practical considerations for clinicians assessing patient-perceived dental attractiveness. For routine evaluations of overall dental attractiveness, mirrors of standard sizes—full-length, medium, or small—can be used interchangeably, as no significant differences in perceived attractiveness were observed between these devices. Among these, the medium-size mirror tended to receive the highest mean ratings, suggesting it may provide a slightly more favourable view, although all standard mirrors remain acceptable for clinical use.

Mirrors with magnification are better suited for assessing fine procedural details, such as tooth surface characteristics or alignment corrections, but may lead to lower perceived dental attractiveness compared with standard mirrors, as magnification can exaggerate minor imperfections. Tooth-shaped mirrors should generally be avoided for aesthetic self-assessment, given their restricted and asymmetric field of view, which may negatively influence patient perception.

Digital reflections via smartphone front-facing cameras can be used as a supplementary tool, reflecting how patients commonly view themselves. However, due to potential image distortion, software enhancements, and colour differences, clinicians should interpret digital assessments cautiously. Furthermore, repeated self-assessment using reflective devices—whether mirrors or digital screens—may be unreliable due to low intra-rater consistency, and should not be relied upon to track changes in dental attractiveness over time.

## Conclusion

Different reflective devices influence patients’ perceived dental attractiveness. Mirrors of varying sizes (full-length, medium, small, and standard pocket) and smartphone digital reflections yielded comparable, generally favourable ratings, whereas magnified and tooth-shaped mirrors produced lower ratings. Intra-rater reliability was mostly poor, indicating that self-assessment using reflections alone is inconsistent.

## References

[1] Frese C, Leciejewski F, Specht R, Wohlrab T, Büsch C, Boemicke W, et al. The dental esthetic screening index: A new tool for assessment of dento-facial esthetics in restorative dentistry. J Esthet Restor Dent. 2019;31(6):572–82. doi: 10.1111/jerd.12522 31483563

[2] Campos LA, Costa MA, Bonafé FSS, Marôco J, Campos JADB. Psychosocial impact of dental aesthetics on dental patients. Int Dent J. 2020;70(5):321–7. doi: 10.1111/idj.12574 32476147 PMC9379174

[3] Bertamini M, Lawson R, Liu D. Understanding 2D projections on mirrors and on windows. Spat Vis. 2008;21(3–5):273–89. doi: 10.1163/156856808784532527 18534104

[4] Berry MV. Inflection reflection: Images in mirrors whose curvature changes sign. Eur J Phys. 2021;42(6).

[5] Asakura N. Experiments with Mirror Reflections. Leonardo. 1990;23(1):71. doi: 10.2307/1578468

[6] Bertamini M, Spooner A, Hecht H. Naive optics: Predicting and perceiving reflections in mirrors. J Exp Psychol Hum Percept Perform. 2003;29(5):982–1002. doi: 10.1037/0096-1523.29.5.982 14585018

[7] Croucher CJ, Bertamini M, Hecht H. Naive optics: understanding the geometry of mirror reflections. J Exp Psychol Hum Percept Perform. 2002;28(3):546–62. doi: 10.1037//0096-1523.28.3.546 12075887

[8] Lawson R, Bertamini M. Errors in judging information about reflections in mirrors. Perception. 2006;35(9):1265–88. doi: 10.1068/p5498 17120845

[9] Bertamini M, Latto R, Spooner A. The Venus effect: people’s understanding of mirror reflections in paintings. Perception. 2003;32(5):593–9. doi: 10.1068/p3418 12854645

[10] Bertamini M, Parks TE. On what people know about images on mirrors. Cognition. 2005;98(1):85–104. doi: 10.1016/j.cognition.2004.11.002 16297677

[11] Lawson R, Bertamini M, Liu D. Overestimation of the projected size of objects on the surface of mirrors and windows. J Exp Psychol Hum Percept Perform. 2007;33(5):1027–44. doi: 10.1037/0096-1523.33.5.1027 17924805

[12] Lawson R. People cannot locate the projection of an object on the surface of a mirror. Cognition. 2010;115(2):336–42. doi: 10.1016/j.cognition.2009.12.013 20096408

[13] Souza F, De Las Casas D, Flores V, Youn SB, Cha M, Quercia D, et al. Dawn of the selfie era: The whos, wheres, and hows of selfies on Instagram. In: COSN 2015 - Proc 2015 ACM Conf Online Soc Networks. 2015. p. 221–31.

[14] Ward B, Ward M, Fried O, Paskhover B. Nasal Distortion in Short-Distance Photographs: The Selfie Effect. JAMA Facial Plast Surg. 2018;20(4):333–5. doi: 10.1001/jamafacial.2018.0009 29494735 PMC5876805

[15] Kajinaka H, Sakamoto Y, Ohara H, Ogata H, Kishi K. Analysis of various distortions in selfies due to differences in smartphone models. J Plast Reconstr Aesthet Surg. 2022;75(1):439–88. doi: 10.1016/j.bjps.2021.09.064 34736852

[16] Suresh N, Sivakumar A. Evaluation of the distortion of photographs using various focal lengths. Bioinformation. 2021;17(9):814–7. doi: 10.6026/97320630017814 35539893 PMC9049081

[17] Shahrul AI, Mohd Shukor N, Norman NH. Does the camera type affect the quality of orthodontic photographs? Mirrorless and smartphone cameras versus digital single lens reflex (DSLR) camera. J Orthod. 2025;52(4):315–25. doi: 10.1177/14653125251358837 40819226

[18] Shahrul AI, Shukor N, Norman NH. Technique for Orthodontic Clinical Photographs Using a Smartphone. Int J Dent. 2022;2022:2811684. doi: 10.1155/2022/2811684 35103062 PMC8800594

[19] Al-Bitar ZB, Hamdan AM, Shqaidef A, Garagiola U, Naini FB. Perception of frontal facial images compared with their mirror images: chirality, enantiomorphic discrimination, and relevance to clinical practice. Maxillofac Plast Reconstr Surg. 2023;45(1):29. doi: 10.1186/s40902-023-00396-4 37639033 PMC10462585

[20] Oranges CM, Schaefer KM, Gohritz A, Haug M, Schaefer DJ. The Mirror Effect on Social Media Self-perceived Beauty and Its Implications for Cosmetic Surgery. Plast Reconstr Surg Glob Open. 2016;4(11):e1088. doi: 10.1097/GOX.0000000000001088 27975012 PMC5142478

[21] Yuen JJX, Saw ZK, Ashari A, Lau MN, Mustapha NMN, Kuppusamy E. Aesthetic perception of gingival display on smiling among laypeople seeking dental treatment. Australas Orthod J. 2023;39(2):136–44.

[22] Marzuki HN, Zahirah I, Lau MN, Kuppusamy E, Mustapha NMN, Ashari A. Likert scale versus the visual analogue scale in evaluating dentofacial aesthetics: a systematic review. Australas Orthod J. 2024;40(1):158–68.

[23] Babeer WA, Bakhsh ZT, Natto ZS. The perception of smile attractiveness to altered vertical position of maxillary anteriors by various groups. Medicine (Baltimore). 2022;101(9):e28660. doi: 10.1097/MD.0000000000028660 35244035 PMC8896490

[24] Ellakany P, Fouda SM, Alghamdi M, Bakhurji E. Factors affecting dental self-confidence and satisfaction with dental appearance among adolescents in Saudi Arabia: a cross sectional study. BMC Oral Health. 2021;21(1):149. doi: 10.1186/s12903-021-01509-z 33757507 PMC7989082

